# Seasonal and geographic differences in treatment-seeking and household cost of febrile illness among children in Malawi

**DOI:** 10.1186/1475-2875-10-32

**Published:** 2011-02-08

**Authors:** Victoria L Ewing, David G Lalloo, Kamija S Phiri, Arantxa Roca-Feltrer, Lindsay J Mangham, Miguel A SanJoaquin

**Affiliations:** 1 Malawi-Liverpool-Wellcome Trust Clinical Research Programme, Blantyre, Malawi; 2 Liverpool School of Tropical Medicine, Liverpool, UK; 3 University of Malawi, College of Medicine, Blantyre, Malawi; 4 London School of Hygiene & Tropical Medicine, London, UK

## Abstract

**Background:**

Households in malaria endemic countries experience considerable costs in accessing formal health facilities because of childhood malaria. The Ministry of Health in Malawi has defined certain villages as hard-to-reach on the basis of either their distance from health facilities or inaccessibility. Some of these villages have been assigned a community health worker, responsible for referring febrile children to a health facility. Health facility utilization and household costs of attending a health facility were compared between individuals living near the district hospital and those in hard-to-reach villages.

**Methods:**

Two cross-sectional household surveys were conducted in the Chikhwawa district of Malawi; one during each of the wet and dry seasons. Half the participating villages were located near the hospital, the others were in areas defined as hard-to-reach. Data were collected on attendance to formal health facilities and economic costs incurred due to recent childhood febrile illness.

**Results:**

Those living in hard-to-reach villages were less likely to attend a formal health facility compared to those living near the hospital (Dry season: OR 0.35, 95%CI0.18-0.67; Wet season: OR 0.46, 95%CI0.27-0.80). Analyses including community health workers (CHW) as a source of formal health-care decreased the strength of this relationship, and suggested that consulting a CHW may reduce attendance at health facilities, even if indicated. Although those in hard-to-reach villages were still less likely to attend in both the dry (OR 0.53, 95%CI 0.25-1.11) and wet (OR 0.60, 95%CI 0.37-0.98) seasons. Household costs for those who attended a health facility were greater for those in HTR villages (Dry: USD5.24; Wet: USD5.60) than for those living near the district hospital (Dry: USD3.45; Wet: USD4.46).

**Conclusion:**

Those living in hard-to-reach areas were less likely to attend a health facility for a childhood febrile event and experienced greater associated household costs. Consulting CHWs was infrequent, but appeared to reduce attendance at a health facility, even when indicated. Health service planners must consider geographic and financial barriers to accessing public health facilities in designing appropriate interventions.

## Background

The link between poverty and malaria has been well established [1-3]. Countries that have successfully eliminated malaria have shown considerable economic growth when compared to other countries that have not [4]. Poor households living in malarious regions struggle to meet the financial cost of repeated bouts of illness [5]. Direct and indirect costs of seeking appropriate health care result in households seeking treatment nearer the home [6]. This occurs in Malawi, where despite the free provision of healthcare through the formal health system, these services are under-utilized and home treatment is common using left over drugs or those obtained from vendors [7]. Consequences of private purchasing of drugs include inappropriate drug selection and dosing [8] potentially leading to death and disability and the promotion of drug resistance [9].

There is considerable concern regarding inequities in health within Africa; improving the health of the population overall is inadequate if groups, such as the very poor, do not benefit [10-13]. In addition to the wealth of the individual, proximity to health facilities is an important determinant of uptake of health services [14,15]. The Malawi government has sought to bring care nearer to patients by establishing a Community Health Worker (CHW) scheme in some villages that have been defined as hard to reach (HTR) either because they are more than 8 km from a public health facility or have reduced accessibility due to rivers or hills [16]. These CHWs operate from small health-posts within the HTR villages. CHW programmes such as this have been advocated as an effective method of reducing barriers to care-seeking [17]. CHWs are involved in delivering health education messages and use a nationally-adapted version of the Integrated Management of Childhood Illness guidelines to treat children aged 2-59 months. However, due to logistical challenges and policies which have emphasized the strict control of ACT prescribing, anti-malarials have not been stocked at health-post level since the switch in first-line anti-malarial from SP to ACTs. CHWs are, therefore, trained to refer all cases of childhood fever to the nearest health facility for treatment.

This study compares communities living in HTR areas with those near the hospital (NTH). The aim was to understand how physical barriers for access to health care and the consequent expenditure incurred by households influence utilization of health facilities.

## Methods

### Study site

This study was carried out in Chikhwawa District, in southern Malawi. This area experiences year round malaria transmission with a peak during the rainy season from December to May. Public health care is provided by the district hospital, 17 health centres and CHWs. There are also a number of private clinics throughout the district. Malaria is a major contributor to the high under five mortality in Malawi and a survey conducted in 2006 found that only 21% of febrile children under the age of five years received a recommended anti-malarial [18].

### Data collection

Two cross-sectional household surveys were conducted in 13 villages: seven were located NTH, defined as within 5 km of the district hospital and six were among villages defined as HTR. To examine seasonal effects, data were collected during both the dry season (June 2009) and the wet season (February 2010). Fieldworkers administered a structured questionnaire to the primary caregivers of eligible children in the local language (Chichewa). Caregivers were eligible to participate in the survey if they had a child less than 10 years and the child had suffered a self-reported febrile episode in the previous two weeks.

The questionnaire had three modules; the first collected details relating to the child and caregiver's characteristics and the child's illness experience. This included questions on the duration of the illness and the caregivers perceived severity of illness, as either mild, moderate or severe. The second module was administered in those instances when health care had been sought and included questions on the sources of care and household direct and indirect costs of seeking care. Direct costs included the costs of travel, consultation, laboratory tests, and treatment, while indirect costs measured the caregiver's time lost from productive activities while caring for the child. The final module collected data relating to socio-economic status by asking about household asset ownership and was based on questions from the Malawi Demographic and Health Survey [19]. The questionnaire was tested during field-staff training and piloted in two villages before each round of the survey, after which minor modifications were made to the questionnaire. Data were collected using Personal Digital Assistants (PDAs) that incorporated internal consistency checks.

### Sampling

This study was conducted as part of a larger study investigating the role of CHWs. Selection of HTR villages was determined by the criteria for the larger study; villages were selected if they were accessible by the study team during both the wet and dry seasons and had a CHW that was actively involved in disease management. A list of HTR villages with CHWs in the Chikhwawa district was provided by the MoH, six fulfilled the study criteria. There are 13 villages within 5 km of the hospital and seven were randomly selected to take part. Within each village, households were randomly selected by field workers finding a central point in the village, spinning a bottle and then walking in the direction the bottle pointed, visiting all houses between the central point and edge of the village. All households to the very edge of the village were visited to ensure that selected households were representative of the village. This process was repeated if insufficient houses were visited.

The study was powered to detect a minimum difference of 40% of recently febrile children in NTH villages attending a health facility compared to 20% of those in HTR villages (power = 90% and α = 0.05 two-sided) during each survey. A design effect of two was estimated, giving a total sample size of 478 recently febrile children in each survey.

### Outcomes

The primary outcome was the proportion of children less than 10 years old who had experienced a febrile episode in the previous two weeks and were taken to a health facility. For the primary analysis, health facilities were defined as private or public clinics and hospitals: CHWs were not included as all febrile children should be referred to a health facility. A second analysis was undertaken to assess attendance at any formal health-care including CHWs. A priori potential confounders were age, maternal education, severity of illness and socio-economic status. The primary economic outcome was the household cost of seeking treatment at a health facility, for those children that reported a febrile episode in the past two weeks. Secondary outcomes included the differences in attendance at a health facility and costs of attending according to season. The proportion of children attending a health facility either on the day of fever onset or the following day and the proportion receiving anti-malarials either on the day of fever onset or the following day were also calculated.

### Analysis of data

Data were cleaned and analysed using Stata version 10 (Stata Corp., College Station, TX). All analyses were adjusted for clustering in the survey design. Unadjusted odds ratios were calculated and then adjusted for a priori potential confounders using a logistic regression model. Mean costs were calculated and censored linear regression was used to assess for differences in costs taking account of incomplete febrile episodes. Values were considered significant if the P-value was less than 0.05 and borderline if less than 0.10.

Both direct and indirect costs incurred by households were included. Indirect costs measure the lost productivity of the caregiver in order to care for the child during the illness episode [20]. Time lost from productive activities to care for the sick child at home was valued as days or half days, while travel time and time at each source of care was measured in days, hours or minutes. Lost productivity was assigned a monetary value based on the Malawi minimum wage for an individual with no professional training and living in a rural area (MK129 per day). All costs were converted from Malawi Kwacha (MK) to US Dollars (USD) using a rate of MK139 to 1USD [21].

Socioeconomic status was measured using the DHS wealth index methodology [19,22]. This method involves using principal components analysis to compute asset indices [23,24]. Each household is scored according to possession of durable goods (such as bicycles), housing quality and sanitary facilities. Households are then ranked into wealth quintiles.

### Ethical considerations

Written consent was obtained from eligible participants in the local language. Consent was given either by signature or thumb-print. Only numerical identifiers were used on data capturing forms. Ethics approval was obtained from the College of Medicine Research and Ethics Committee, Malawi and from the London School of Hygiene and Tropical Medicine Ethics Committee.

## Results

A total of 1,181 households were surveyed in the dry season and 1,397 in the wet season. None of the eligible households refused to participate. Forty-one percent (482/1,181) of households in the dry season and 35% (484/1,397) in the wet had at least one child under the age of 10 years who had experienced a febrile event in the two weeks prior to the survey and for whom a suitable caregiver could be found. At the time of the surveys, 47 children in the dry season and 60 during the wet were still suffering from reported fever. Forty-five percent (186/416) of individuals taking part in the survey during the wet season, had previously taken part in the dry season survey.

Table 1 shows the background characteristics according to distance to hospital and season. The distribution of age, sex and severity of illness was similar between HTR and NTH villages and across season. However, mothers of those living in HTR villages had attained lower levels of education compared to those NTH (Dry: P = 0.06; Wet P = 0.03). Households in HTR villages tended to occupy the lower wealth quintiles compared to those NTH (Dry: P = 0.002; Wet: P = 0.01). Eighty-six percent (212/246) of the caregivers in HTR villages were farmers compared to 64% (168/263) of those NTH (wet season data available only). The proportion of recently febrile children who were less than five years were similar across seasons (Dry: 63%: Wet: 65%). Only one death occurred from reported recent febrile illness (wet season). In children seeking health facility care, mean illness length was longer for those in HTR villages than for those NTH (Dry: 5.8 vs 4.9 days, P = 0.090; Wet: 5.6 vs 4.8 days, P = 0.028). However illness length did not differ between seasons.

**Table 1:** Association between participant background characteristics and distance to formal health facility

	Dry season			Wet season		
**Background characteristics**	**≤ 5 km** ** from ** **Hospital** **(%) n = 269**	**Hard to** ** Reach** ** (%) n = 262**	**P^a^**	**≤ 5 km ** **from ** **Hospital** **(%) n = 263**	**Hard to** ** Reach** **(%) n = 246**	**P^a^**

**Child Sex**						
Male	131 (49)	123 (47)		138 (52)	126 (51)	
Female	138 (51)	139 (53)	0.57	125 (48)	120 (49)	0.75

**Child Age**						
<5 years	176 (65)	160 (61)		175 (67)	157 (64)	
≥5 years	93 (35)	102 (39)	0.12	88 (33)	89 (36)	0.48

**Maternal Education**						
None	63 (23)	87 (33)		68 (26)	87 (35)	
Primary	153 (57)	148 (57)		159 (60)	145 (59)	
Secondary +	53 (20)	27 (10)	0.06	36 (14)	14 (6)	0.03

**Wealth Quintile**						
1 (Lowest)	29 (11)	76 (29)		31 (12)	68 (28)	
2	41 (15)	66 (25)		47 (18)	51 (21)	
3	41 (15)	65 (25)		48 (18)	63 (25)	
4	68 (25)	38 (15)		57 (22)	44 (18)	
5 (Highest)	90 (34)	17 (6)	<0.01	80 (30)	20 (8)	0.01

**Illness Severity^b^**						
Mild	155 (58)	134 (51)		132 (50)	111 (45)	
Moderate	81 (30)	78 (30)		93 (35)	88 (36)	
Severe	31 (12)	49 (19)	0.11	38 (15)	46 (19)	0.24

^a^F test for heterogeneity or trend, with adjustment for clustering in survey design

^b^Missing data - four individuals did not know or remember the severity of the illness

In general, those in HTR villages had access to lower quality sanitation and owned fewer possessions. Absence of toilet facilities was more common among those in HTR villages (Dry: 14%; Wet: 12%) compared to those NTH (Dry: 5%; Wet: 8%). However, more of those in HTR villages owned agricultural land (Dry: 97%; Wet: 99%) compared to those NTH (Dry: 76%; Wet: 84%).

### Treatment-seeking

Table 2 shows attendance to a health facility by background characteristics. In the dry season 48% of children in HTR villages were taken to a health facility compared to 69% of children living NTH. During the wet season this increased slightly to 53% of those in HTR villages and 70% of those living NTH. Children in HTR villages were less likely to attend a health facility for a recent childhood febrile event in both the dry (OR 0.35, 95%CI 0.18 - 0.67, P = 0.004) and the wet (OR 0.46, 95%CI 0.27-0.80, P = 0.01) seasons. Attendance at a health facility was less common among those over five years compared to those under five in the dry season (OR 0.49, 95%CI 0.31 - 0.79, P = 0.005), however this association was not significant in the wet season. Children suffering from more severe illnesses were more likely to be taken to a health facility in both the dry (moderate illness: OR1.80, 95%CI1.26-2.57; severe illness: OR3.97, 95%CI2.25-7.02, P < 0.001) and wet (moderate illness OR3.08, 95%CI2.03-4.67; severe illness OR4.49, 95%CI2.33-8.65, P < 0.001) seasons.

**Table 2:** Odds ratio for attending a formal health facility by background characteristics and season

	Dry Season		Wet Season	
**Background characteristics**	**N (%)**	**Odds Ratio (95% CI)^a^**	**N (%)**	**Odds Ratio (95% CI)^a^**

**ATTENDED FACILITY**				

**Village of residence**				
≤5 km from Hospital	185 (69)	1	184 (70)	1
Hard to Reach	126 (48)	0.35 (0.18 - 0.67)^b^	133 (53)	0.46 (0.27-0.80)^b^

**Child Age**				
<5 years	218 (65)	1	218 (65)	1
5 - 10 years	93 (48)	0.49 (0.31 - 0.79)^b^	99 (56)	0.69 (0.40-1.20)

**Maternal Education**				
None	82 (55)	1	83 (53)	1
Primary	176 (58)	1.09 (0.74 - 1.62)	200 (66)	1.32 (0.77-2.28)
Secondary +	53 (66)	1.32 (0.69 - 2.44)	34 (67)	1.29 (0.54-3.08)

**Socio-economic Status**				
Poorest quintile	59 (56)	1	56 (56)	1
Quintile increase	252 (59)	0.90 (0.75 - 1.10)	261 (64)	1.03 (0.90-1.18)

**Illness Severity**				
Mild illness	147 (51)	1	117 (48)	1
Moderate illness	101 (64)	1.80 (1.26 - 2.57)	133 (73)	3.08 (2.03 - 4.67)
Severe illness	61 (76)	3.97 (2.25 - 7.02)^c^	66 (79)	4.49 (2.33 - 8.65)^c^
**ATTENDED FACILITY WITHIN 24 HOURS**				

**Village of residence**				
≤5 km from Hospital	147 (55)	1	151 (57)	1
Hard to Reach	83 (32)	0.32 (0.16 - 0.62)^b^	109 (43)	0.53 (0.32 - 0.88)^d^
**ATTENDED FACILITY OR COMMUNITY HEALTH WORKER**				

**Village of residence**				
≤5 km from Hospital	187 (70)	1	184 (70)	1
Hard to Reach	155 (59)	0.53 (0.25-1.11)^e^	151 (61)	0.60 (0.37-0.98)^f^

All analyses adjusted for survey design

^a^Odds Ratio adjusted for a priori confounders; age, maternal education, socio-economic status and illness severity

^b^P≤0.01, ^c^P < 0.001, ^d^P = 0.02, ^e^P = 0.09, ^f^P = 0.04

Time between onset of illness and attendance at a health facility was examined. In the dry season 32% (83/262) of those in HTR villages compared to 55% (147/269) of children living NTH attended a health facility on the day of fever onset or the next day (P = 0.003). In the wet season 43% (109/246) living in HTR villages compared to 57% (151/263) of those living NTH attended a health facility either on the day of fever onset or the next day (P = 0.02). Children in HTR villages who attended a health facility were more likely to do so on the day of fever onset or the next day during the wet season compared to the dry season (OR 2.24, 95%CI 1.30 - 3.86, P = 0.01).

The data were also analysed to assess the relationship between village of residence and attendance to any formal health-care including CHWs. Those in HTR villages were still less likely to attend any formal health-care compared to those living NTH in both the dry (OR 0.53, 95%CI 0.25-1.11, P = 0.09) and wet (OR 0.60, 95%CI 0.37-0.98, P = 0.04) seasons. However, including CHWs in the analysis decreased the strength of the relationship between location and care-seeking. Eighty-nine percent (49/55) of individuals who sought care from a CHW did not go on to attend a formal health facility.

### Household cost

Table 3 shows the mean costs of a childhood febrile episode among those who attended a health facility by distance to hospital and season. In the dry season, the mean total cost of a childhood febrile episode was USD5.24 for those in HTR and USD3.45 for those NTH villages (P = 0.03). In the wet season, the mean total cost was USD5.60 for those in the HTR villages and USD4.46 for children NTH (P = 0.12).

**Table 3:** Mean cost of seeking care for a childhood febrile episode by distance to formal health facility

	Dry Season			Wet Season		
		
	<5 km from Hospital (n = 269) (USD)	Hard to Reach (n = 262) (USD)	P^a^	<5 km from Hospital (n = 263) (USD)	Hard to Reach (n = 246) (USD)	P^a^
		
Direct Costs^b^						
Travel	0.17	0.31^d^	0.03	0.28	0.89^d^	0.03
Consultation	0.01	0.02	0.43	0.05	0.00	0.18
Treatment	0.02	0.05	0.24	0.07	0.06	0.72
Total Direct Costs	0.20^e^	0.38^f^	0.04	0.40^e^	0.95^f^	0.08

**Indirect Costs^c^**						

Travel time	0.07	0.15	0.11	0.08	0.16	<0.001
Time at facility	0.78	0.69	0.58	0.86	0.67	0.27
Time caring at home	2.40	4.01	0.02	3.13	3.82	0.12
Total Indirect Costs	3.25	4.86	0.07	4.06	4.65	0.32

**Total costs**	3.45	5.24	0.03	4.46	5.60	0.12

^a^T test for the difference in means using censored linear regression with adjustment for clustering in survey design and confounding of child age, severity of illness and socioeconomic status

^b^Out of pocket expenses

^c^Cost of time losses

^d^P = 0.04 for the difference between seasons for those living in hard to reach villages

^e^P = 0.06 for the difference between seasons for those living <5 km of district hospital

^f^P = 0.05 for the difference between seasons for those living in hard to reach villages

Travel costs made the largest contribution to direct cost, and for those living in HTR villages these increased significantly between the dry and wet seasons (Dry: USD0.31; Wet: USD0.89, P = 0.04). Direct costs were greater for those living in HTR compared to NTH villages in both the dry (USD0.38 vs USD0.20, P = 0.04) and wet (USD0.95 vs USD0.40, P = 0.08) seasons. Direct costs were greater in the wet season for both those in HTR (P = 0.05) and NTH (P = 0.06) villages. Indirect costs represented the main economic burden for households. These were USD4.86 for those in HTR villages and USD3.25 for those NTH in the dry season and USD4.65 for those in HTR villages and USD4.06 for those NTH in the wet season. Most indirect costs were due to 'time caring at home'. The cost of time spent caring was greater for those in HTR (USD2.40) compared to NTH (USD4.01) villages in the dry season (P = 0.02), but did not differ significantly by village in the wet season (P = 0.12).

## Discussion

This study compares the utilization and household costs of attending health facilities for febrile illness in two geographically defined communities: households within 5 km of the hospital (NTH) and households that are 8 km from a health facility and considered hard to reach (HTR). Those in HTR villages were less likely to attend a health facility than those NTH and experienced greater costs in seeking treatment. Costs associated with travel to the health facility were significantly greater for those in HTR areas, and also increased markedly during the wet season. The costs associated with a febrile illness increase substantially when the indirect costs associated with the caregiver's loss of productive activity during the illness episode are also taken into consideration.

These findings are based on the caregivers' responses to questions relating to a febrile illness episode, which is an accepted methodology in economic studies [5,25,26]. The measurement of severity of the illness was dependent on the caregiver's assessment, though previous studies have shown that caregivers are able to recognize both mild and severe signs of illness [27]. To reduce reporting error relating to the caregiver's account of the fever episode and the costs involved, the recall period was limited to the previous two weeks. In economic studies, it is advisable to estimate the indirect as well as direct costs [28]. This is not without challenges, particularly in non-wage settings such as this. In the absence of salary data, it is common practice to value caregiver's lost productivity using the minimum wage [26]. The inclusion criteria for this study included year round accessibility of HTR villages to the study team. Less accessible villages might make even less contact with formal health facilities and may experience greater costs, particularly in the form of travel costs and time.

Each of the HTR villages taking part in this study have been allocated a CHW. The CHW health posts are placed within the communities, so direct and indirect costs of attendance are minimal. CHWs working in HTR villages in Malawi treat a range of illnesses, but do not currently treat malaria. Instead all febrile children should be referred to a health facility. This study found attendance to CHWs to have been infrequent, despite their proximity to community members, and the majority of those attending a CHW did not go on to attend a health facility. The current CHW referral programme should be assessed both in terms of establishing reasons for the low uptake of services, and lack of referral visits to health facilities. The inclusion of Artemisinin-based Combination Therapy (ACT) in the services provided by CHWs is being considered by the MoH. The inability of CHWs to prescribe anti-malarials is recognized by communities and is likely to adversely impact attendance at CHWs. Making antimalarials available would increase utilization of CHWs and considerably improve access to prompt appropriate treatment for the poorest and most at risk members of the population, whilst reducing the costs of accessing care.

As previously found in Malawi, perception of severity of illness was associated with higher health facility attendance [29], and for those living NTH this was more marked in the wet than the dry season. Care-seeking delays were more common among those living in HTR villages during both seasons. However those in HTR villages that sought care, did so sooner during the wet compared to the dry season, despite poor accessibility due to the rains. This may be due to caregivers' knowledge that fevers during the wet season are more likely to be malaria. The balance of risks for those in HTR villages of the severity of the child's illness, the cost of attending for care and the potential costs of not attending is complex and deserves in-depth investigation. The HTR villages taking part in this study were accessible to the study team and this pattern of increased promptness of treatment-seeking during the wet season may not be seen in less accessible villages.

Despite free healthcare provision at public health facilities, residents of the Chikhwawa district still experience considerable costs in accessing these facilities and costs are greater amongst those in HTR areas than villages located around the hospital. This study investigated single febrile episodes occuring in each of the wet and dry seasons. However children in the Chikhwawa district experience repeated febrile episodes and costs may be considerably greater if care is sought for each febrile episode. The definition of HTR is used in health system planning in Malawi, however this is the first study to investigate the impact of living in such areas on the household economic cost of childhood febrile illness. This study shows that even within a poor, rural population there are differences in wealth, access to formal health facilities and prompt treatment of childhood fever according to geographic location.

## Conclusion

Those living in HTR areas were less likely to attend a health facility for a childhood febrile event and experienced greater associated household costs. Attendance to CHWs was infrequent but appeared to reduce attendance at a health facility, even when indicated. Since CHWs do not stock antimalarials this may have reduced the proportion of children receiving appropriate care. Health service planners must consider geographic and financial barriers to accessing public health-facilities in designing appropriate interventions.

## Competing interests

The authors declare that they have no competing interests.

## Authors' contributions

VE and MS designed the survey with assistance from LM. VE supervised survey conduction, undertook the data analysis and drafted the paper. LM, MS and DL provided study supervision. LM and MS assisted with data analysis. KP and AR provided guidance throughout the study process. All authors were involved in revising the paper for publication.
